# Oxidative Imbalance as a Crucial Factor in Inflammatory Lung Diseases: Could Antioxidant Treatment Constitute a New Therapeutic Strategy?

**DOI:** 10.1155/2021/6646923

**Published:** 2021-02-09

**Authors:** Tatiana Victoni, Emiliano Barreto, Vincent Lagente, Vinicius F. Carvalho

**Affiliations:** ^1^University of Lyon, VetAgro Sup, APCSe, Marcy l'Étoile, France; ^2^Laboratory of Cell Biology, Federal University of Alagoas, Maceió, AL 57072-900, Brazil; ^3^NuMeCan Institute (Nutrition, Metabolism and Cancer), INSERM, INRAE, CHU Rennes, Univ Rennes, Rennes, France; ^4^Laboratório de Inflamação, Instituto Oswaldo Cruz, Fundação Oswaldo Cruz, Rio de Janeiro, RJ 21045-900, Brazil

## Abstract

Inflammatory lung disease results in a high global burden of death and disability. There are no effective treatments for the most severe forms of many inflammatory lung diseases, such as chronic obstructive pulmonary disease, emphysema, corticosteroid-resistant asthma, and coronavirus disease 2019; hence, new treatment options are required. Here, we review the role of oxidative imbalance in the development of difficult-to-treat inflammatory lung diseases. The inflammation-induced overproduction of reactive oxygen species (ROS) means that endogenous antioxidants may not be sufficient to prevent oxidative damage, resulting in an oxidative imbalance in the lung. In turn, intracellular signaling events trigger the production of proinflammatory mediators that perpetuate and aggravate the inflammatory response and may lead to tissue damage. The production of high levels of ROS in inflammatory lung diseases can induce the phosphorylation of mitogen-activated protein kinases, the inactivation of phosphoinositide 3-kinase (PI3K) signaling and histone deacetylase 2, a decrease in glucocorticoid binding to its receptor, and thus resistance to glucocorticoid treatment. Hence, antioxidant treatment might be a therapeutic option for inflammatory lung diseases. Preclinical studies have shown that antioxidants (alone or combined with anti-inflammatory drugs) are effective in the treatment of inflammatory lung diseases, although the clinical evidence of efficacy is weaker. Despite the high level of evidence for the efficacy of antioxidants in the treatment of inflammatory lung diseases, the discovery and clinical investigation of safer, more efficacious compounds are now a priority.

## 1. Introduction

Airway inflammation is now acknowledged to have a causative role in the pathophysiology of several major lung diseases, including asthma, chronic obstructive pulmonary disease (COPD), acute respiratory distress syndrome (ARDS), allergic rhinitis, cystic fibrosis, cough, emphysema, and lung fibrosis. The development of inflammation is a complex series of events that involves the release of proinflammatory cytokines and then the recruitment of polymorphonuclear neutrophils, eosinophils, and/or mononuclear cells in the lung tissue [1]. For example, the chronic inflammation in COPD involves the infiltration of the main types of inflammatory cell (including neutrophils, monocytes/macrophages, and lymphocytes) into the airway and the lung tissue; the cells can be detected in bronchoalveolar fluid and induced sputum [2]. Nevertheless, there are a number of disease-specific differences in the inflammatory pathophysiological processes. For example, chronic airway inflammation of the central and peripheral airways in chronic severe asthma is typically characterized by the same pathological features as in mild-to-moderate persistent asthma, with increased numbers of activated T lymphocytes (particularly CD4^+^ Th2 cells) and (sometimes) eosinophils and mast cells [3]. The most notable difference between chronic severe asthma and mild-to-moderate persistent asthma is the elevated neutrophil count in the former context. In stable COPD, chronic airway inflammation of both central and peripheral airways is characterized by increased numbers of T lymphocytes (particularly CD8^+^ cells), macrophages, and neutrophils. The macrophage and neutrophil counts increase with the disease progression and severity.

It is generally acknowledged that persistent chronic inflammation contributes to both parenchyma remodeling and bronchial remodeling [4]. Remodeling has been observed in central airways, distal airways, and the lung parenchyma. This process of structural changes involves hyperplasia of the airway epithelial cells, thickening of the reticular basement membrane, deposition of collagen, peribronchial fibrosis, airway epithelial-to-mesenchymal transition, and bronchial smooth muscle cell hyperplasia [5]. The inflammatory reaction is followed by damage to the basement membrane through at least two different mechanisms: the production of reactive oxygen species (ROS) and the synthesis of proteases. In a healthy lung, the intactness of basement membrane reflects the dynamic balance between synthesis and degradation of its components—mainly proteases and antiproteases. These enzymes are synthesized constitutively by mesenchymal cells (such as fibroblasts, macrophages, endothelial cells, and epithelial cells) and inflammatory cells (such as monocytes/macrophages, neutrophils, and eosinophils) [6].

When treating respiratory disease, the main objectives are to reduce symptoms and prevent and decrease the number of exacerbations by reducing inflammation. Although today's treatments (e.g., a combination of a corticosteroid anti-inflammatory and a *β*2 agonist bronchodilator) achieve these goals to a certain extent in asthma, it is still not possible to prevent a decline in lung function. Moreover, the efficacy of anti-interleukin (IL)-5 and anti-IL-13 antibodies in severe asthma clearly demonstrates the need for both careful patient phenotyping and the need for reliable biomarkers of patient phenotypes and drug efficacy [7, 8].

Furthermore, it is thought that specific cytokines control the corticosteroid insensitivity, fibrosis, and remodeling observed in COPD, ARDS, and fibrosis. Hence, targeting these cytokines might usefully reverse these changes. Although there is a large body of literature data on the roles of various cytokines in inflammatory disorders (except asthma), the effect of specific cytokine blockade in inflammatory respiratory disorders has not been extensively investigated. The list of cytokines and chemokines implicated in the many facets of COPD pathogenesis is very long. Some have been identified in genome-wide association studies of COPD, lung function, and the complications of COPD. The two largest studies published to date involved the use of anti-TNF-*α* and CXCL8 (IL-8) blocking antibodies, respectively; neither provided clinical benefit [9]. Specific groups of COPD patients should be targeted with a specific anticytokine therapy if there is evidence of (i) high expression of that cytokine and (ii) potentially responsive clinical features of disease [10]. The effects of anti-IL-5 and anti-IL-5R antibodies elicited a beneficial effect against the risk of exacerbation in phenotype patients. Thus, patients can be taken off a treatment if it is ineffective to reduce the risk of any possible side effects [11].

In contrast, several studies have suggested that inflammasomes (and particularly the NLRP3 inflammasome) might be involved in the pathogenesis of fibrotic lung diseases, including idiopathic pulmonary fibrosis (IPF) and diseases elicited by known environmental exposure (e.g., asbestosis and silicosis) [12]. More recent data in mice favor a role for inflammasome-independent induction of IL-1*β* in driving smoke-induced inflammation [10]. This is in line with a recent study that showed that a monoclonal antibody neutralizing IL-1*β* was ineffective in the treatment of stable COPD [11].

Recently, literature data have suggested that the combination of oxidative stress and chronic inflammation in the lungs is associated with aging and may contribute to age-related immune dysfunction and the risk of death in older adults infected by respiratory viruses such as severe acute respiratory syndrome coronavirus 2 [13]. The objective of the present chapter is to assess the involvement of oxidative imbalance and ROS in the development of respiratory diseases and review new potential treatments or adjunct therapies based on antioxidant compounds.

## 2. The Role of ROS in the Development of Lung Disease

ROS are ions or small molecules that contain oxygen and an unpaired electron conferring high reactivity. In mammals, ROS are produced by endogenous prooxidant enzymes such as nicotinamide adenine dinucleotide phosphate (NADPH) oxidase (NOX), xanthine oxidase (XO), peroxisomal enzymes, and cytochrome P-450 (CYP450) [14]. A difference between ROS production and removal results in a redox imbalance, which can be controlled by treatment with exogenous antioxidants such as vitamins C and E, polyphenols, carotenes, flavonoids, omega-3 fatty acids, and N-acetylcysteine (NAC). Patients with respiratory diseases like asthma and COPD show elevated levels of ROS production and oxidative stress—suggesting that their endogenous antioxidants may not be sufficient to prevent oxidative damage by cigarette smoke exposure [6, 15–17]. Furthermore, the inflammatory processes associated with the recruitment and activation of phagocytic cell types (namely, neutrophils and mononuclear cells) may also have a role in generating endogenous oxidative stress. Oxidants are known to interfere with the protease/antiprotease imbalance, leading to airway remodeling and emphysema [6, 18]. Indeed, components of the lung matrix (such as elastin and collagen) can be directly degraded by oxidants. We previously demonstrated the inability of phagocytes from p47phox^−/−^ knockout mice to produce large quantities of ROS via the NOX pathway, which inhibits the development of bleomycin-induced pulmonary fibrosis. This inhibition is associated with changes in IL-6 production and in the molar ratio of matrix metalloproteinase 9 (MMP-9) to tissue inhibitors of metalloproteinases (TIMP-1)—both of which are probably key factors in airway remodeling and fibrosis [19].

Oxidative imbalance is reportedly an important factor in the pathogenesis of asthma [20], COPD [16], acute lung injury [21], pulmonary fibrosis [21], and COVID-19 [13]. Cells and tissues are steadily exposed to oxidants generated by endogenous metabolic reactions (e.g., via mitochondrial respiration or phagocyte activation) or absorbed from the environment (e.g., air pollutants and cigarette smoke) [22].

Under physiological conditions, the level of intracellular oxidant species is dynamically stabilized by enzymatic and nonenzymatic cellular processes that produce or eliminate ROS [23]. Enzymatic antioxidants work by breaking down and removing free radicals: the main enzymes are ascorbate peroxidase (APx), glutathione peroxidase (GPx), metallothionein-3 (MT-3), ferritin heavy chain (FHC), dihydrodiol dehydrogenase (DD), catalase (CAT), and superoxide dismutase (SOD) [24]. Intrinsic nonenzymatic antioxidants work by interrupting free radical chain reactions and notably include metal-binding proteins, glutathione, uric acid, melatonin, bilirubin, and polyamines [25].

An oxidative imbalance results in the generation of ROS and intracellular signaling events that trigger the production of proinflammatory mediators and thus stimulate the development of histological changes in the lung. Although the oxidant agents and mechanisms are highly diverse, several common features have emerged. It is well established that the accumulation of highly reactive molecules causes generalized damage to DNA and increases lipid peroxidation and protein carbonyl formation in lung tissue [26]. Thus, ROS directly impact cell proliferation, cell differentiation, immune function, and vasoregulation—all of which are involved in the progression of lung diseases. These effects are exerted through distinct enzymatic complexes (such as kinases, G protein-coupled receptors, ion channel function, and transcription factors) and lead to onset and progression of lung diseases [27].

One of the first consequences of an oxidative imbalance is lipid degradation, resulting from reactions between free radicals and lipids containing carbon-carbon double bonds (especially polyunsaturated fatty acids). If this reaction is not limited, it can permanently damage cell membranes due to the accumulation of lipid peroxidation end products [28]. Levels of the end product malondialdehyde are predictive of COPD exacerbations [29]. Furthermore, malondialdehyde levels are positively correlated with increased expression of Toll-like receptor 4 (TLR4) and factor nuclear kappa B (NF-*κ*B)—signaling pathways involved in lung diseases [30]. This relationship is further illustrated by data from animal experiments in which blockade of the TLR4/NF-*κ*B pathway restored both functional and morphological features of the lungs in asthma [31], COPD [32], acute lung injury [30], and pulmonary fibrosis [33] models.

A growing body of research data has evidenced the relationship between ROS and classical intracellular signaling pathways, such as those involving mitogen-activated protein kinase (MAPK), nuclear factor erythroid 2-related factor 2- (Nrf2-) ARE, phosphoinositide-3-kinase- (PI3K-) Akt, and Ca^2+^ in lung diseases [33, 34]. Even though it is not fully clear how ROS activate these pathways, the oxidative imbalance has been directly implicated in the pathogenesis of asthma [35], COPD [36], and IPF [37].

Over the last decade, a body of scientific data has highlighted the involvement of other important molecular targets in the pathogenesis of pulmonary diseases, such as endoplasmic reticulum (ER) stress (the accumulation of misfolded proteins in the ER), the inflammasome, and the P2X7 purinergic receptor. The ER has a major role in the synthesis, folding, and structural maturation of many proteins made in the cell [38]. When misfolded proteins accumulate in the ER, the intracellular signaling pathway called the unfolded protein response (UPR) induces a set of transcriptional and translational events that restore ER homeostasis [39]. If high levels of ER stress persist, a terminal UPR program prompts cells to increase ROS production; this disturbance leads to self-destruction of the cell [40]. All the events triggered by UPR have been linked to the pathogenesis of distinct respiratory conditions, including cystic fibrosis, COPD, asthma, IPF, and lung infections [17, 38, 40].

Inflammasomes are intracellular multiprotein innate immune complexes. Once activated, the inflammasome's enzymatic activity is mediated by the recruitment and activation of caspase-1 [41]. These multiprotein complexes can influence oxidative imbalance and have emerged as an important regulator of lung disease [42]. Activation of the best-studied inflammasome (the NLR protein NLRP3/NALP3) triggers the production of proinflammatory mediators and ROS associated with lung injury [43–45]. The involvement of oxidative imbalance in this mechanism is further emphasized by the antioxidant-induced inhibition of inflammasome activation—suggesting that redox signaling is involved in NLRP3/NALP3 activation [46].

The P2X7 purinergic receptor (P2X7R) is an important ATP-responsive immunomodulator. It has been implicated in the development of inflammatory respiratory diseases [47]. The receptor's key role has been characterized in models of pulmonary fibrosis, lung inflammation, asthma, and COPD [48]. P2X7R is constitutively expressed by many cell types (including respiratory tract epithelial cells) and participates in the release of proinflammatory cytokines, collagen deposition in the lung, activation of the NLRP3-inflammasome pathway, and ROS production. These data highlight P2X7R as a potential therapeutic target in lung disease. Indeed, P2X7R antagonists reduce neutrophil infiltration and proinflammatory cytokine levels in acute lung injury [49, 50]. Various P2X7R antagonists are currently under clinical development. Furthermore, other purinergic receptor (P2R) agonists and antagonists have been a drug candidate for the treatment of COPD and chronic cough; in particular, an antagonist at P2X2/3R antagonists and some of (P2R) agonists and antagonists might also be relevant for the treatment of other lung diseases [51, 52].

## 3. The Impact of ROS on Glucocorticoid Resistance in Inflammatory Lung Diseases

Local and systemic treatments with glucocorticoids are not effective in some patients with inflammatory lung disease—especially those with severe disease or those exposed to respiratory viruses, cigarette smoke, or air pollution [53, 54]. In clinical terms, glucocorticoid resistance is defined as a failure to raise forced expiratory volume in the first second (FEV1) by 15% following a 7-day course of oral corticosteroid at a daily prednisolone dose equivalent of 20 mg. Although these patients do not benefit from corticosteroid therapy, they nevertheless experience the characteristic adverse drug reactions linked to systemic glucocorticoid treatment [55].

Several mechanisms have been linked to the development of corticosteroid resistance, including immune-mediated dysregulation of cytokines, excessive activation of mitogen-activated MAPK, activating peptide-1 (AP-1) and factor nuclear kappa B (NF-*κ*B), defects in the ability of the glucocorticoid receptor (GR) to bind the drug and translocate into the nucleus, amplified GR*β* isoform expression, and abnormal histone acetylation [54]. The Th17 immune response appears to have a key role in steroid resistance in inflammatory lung diseases because there is a correlation between Th17 cell-induced elevation of IL-17 and steroid-resistant disease through neutrophil accumulation [55]. Nevertheless, merely preventing neutrophilic inflammation may not be effective in corticosteroid-resistant lung diseases because the neutralization of TNF-*α* (a powerful inducer of neutrophil chemotaxis) did not improve FEV1 in patients with severe asthma after high-dose corticosteroid treatment [56].

Some of the cytokines produced in excess by patients with severe asthma (including IL-2, IL-4, and IL-13) enhance p38MAPK activity. The MAPK-induced phosphorylation of serine 134 on the GR leads to steroid resistance by impeding nuclear translocation, protein stabilization, and DNA binding [57–59]. We showed previously that repeated allergen exposure induces glucocorticoid-insensitive asthma, increased phosphorylation of GATA-3 and p38MAPK, and reduced GR availability in A/J mice [60].

The inactivation of GR by MAPKs decreases the receptor's ability to induce histone acetylation, which in turn prevents the interaction with proinflammatory transcription factors AP-1 and NF-*κ*B [61]. Additional steroid resistance mechanisms include the reduction of histone deacetylase (HDAC) 2 activity by phosphoinositide 3-kinase (PI3K) *δ* [62]. Lastly, numbers of inflammatory cells expressing GR*β* isoform immunoreactivity are higher in glucocorticoid-resistant patients than in glucocorticoid-sensitive patients. Although the *β* isoform of the GR only differs from the *α* isoform at its carboxyl-terminal region, this is enough to prevent glucocorticoids from binding. Nevertheless, the GR*β* is able to bind to the glucocorticoid response element—even in the absence of the ligand—but cannot activate the promoter of glucocorticoid-responsive genes. When GR*β* is strongly expressed, activation of GR*α* by glucocorticoids does not therefore result in gene transactivation; consequently, glucocorticoid resistance is observed [63].

Levels of ROS and their metabolites are higher in patients with COPD and severe asthma than in healthy subjects [64, 65]. Furthermore, the *in vitro* activation of peripheral blood neutrophils or mononuclear cells obtained from patients with COPD or asthma increased ROS production and serves as a severity marker for these two inflammatory lung diseases [66–69]. Therefore, elevated ROS production in these diseases might be linked to glucocorticoid resistance. The creation of prooxidant cellular environment *in vitro* (achieved by treatment with tertiary butyl hydroperoxide, an organic hydroperoxide) prevented glucocorticoids from inhibiting IL-8 production by macrophages [69]. Furthermore, H_2_O_2_ also decreases glucocorticoid response element activation in human lung epithelial BEAS-2B cells *in vitro*—suggesting that glucocorticoid resistance had been induced [70].

In a murine model of asthma, ozone-induced exacerbation of asthma is accompanied by elevated levels of oxidative stress, IL-17 production, airway neutrophilia, and the development of glucocorticoid resistance. This glucocorticoid insensitivity on the murine asthma model was associated with an increase in the phosphorylation of p38MAPK and the reduction of MKP-1 activation. In addition, the inhibition of MAPK by SB239063 in this model reversed the ability of glucocorticoid to inhibiting inflammatory response and airway hyperresponsiveness through the reduction in p38MAPK phosphorylation and increase in MKP-1 activation [71]—suggesting that ROS may provoke corticosteroid resistance by excessive activation of p38MAPK. Indeed, *in vitro* ROS-induced glucocorticoid resistance in monocytes and macrophages was related to an increase in p38MAPK phosphorylation and a reduction in HDAC activity, respectively [69, 72].

Nitrosylation and oxidation of the GR reduce the glucocorticoid binding, nuclear translocation, and DNA binding [73, 74]. The ROS-induced impaired nuclear translocation of GR appears to be mediated by the oxidation of the receptor's Cys-481 residue [74]. Furthermore, nitrosylation can modulate GR expression. For instance, neuronal nitric oxide synthase is an endogenous inhibitor of GR expression in the hippocampus [75]. Nevertheless, this action is subject to debate because inhaled NO restored endotoxin-induced downregulation of the GR expression in the lung, liver, and kidney [76]. In severe asthma and COPD, inducible nitric oxide synthase is upregulated [21]. The high resulting NO production might explain the decrease in glucocorticoid responsiveness. Although this mechanism might be relevant in glucocorticoid-resistant patients, selective inducible nitric oxide synthase inhibitors have not yet been evaluated in the clinic.

Tyrosine nitration of HDAC2 results in its inactivation, ubiquitination, and degradation [77]. ROS also increased the activity of PI3K*δ*, which leads to the phosphorylation and inactivation of HDAC2 [62]. Furthermore, H_2_O_2_ induced steroid insensitivity and reduced *β*2 adrenoceptor-dependent cAMP production via the inhibition of PI3K*δ* signaling in U937 cells *in vitro* [78]. HDAC2 inactivation is related to glucocorticoid insensitivity in COPD patients [79, 80], suggesting that ROS have a fundamental role in the development of glucocorticoid resistance.

## 4. Could Antioxidant Treatment Be Effective in Lung Diseases?

As discussed above, oxidative imbalance and the generation of ROS are known to contribute to the pathogenesis of a number of important lung diseases. Hence, several therapeutic strategies have been suggested for eliminating ROS and/or restoring the redox balance. Here, we summarize current knowledge on ROS and oxidative imbalance as therapeutic targets. Antioxidant drugs can be divided into three large groups, as a function of their mechanism of action: (i) those that functionally enhance endogenous antioxidant enzymes such as SOD, CAT, and GPx, which accelerates the conversion and inactivation of free radicals; (ii) nonenzymatic scavengers of excess free radicals and lipid peroxyl radicals, which keep the cell membrane intact; and (iii) drugs with other mechanisms.

### 4.1. Antioxidant Drugs Can Enhance the Function of the Endogenous Antioxidant Enzyme System

NAC is a classical antioxidant that provides cysteine for the increased intracellular production of glutathione. In fact, NAC is a pleiotropic drug with various pharmacologic characteristics. It was developed as a mucolytic agent, since it breaks down mucin disulfide cross-links, reduces the viscosity of mucus and lung secretions, and reestablishes oxygen saturation in the blood [77]. NAC also directly inactivates reactive electrolytes and free radicals in a nonenzymatic manner and maintains the oxidant/antioxidant balance in cells. At higher doses, NAC reduces the formation of proinflammatory cytokines, such as IL-9 and TNF-*α* [81] [82]. For years, it was believed that NAC's beneficial effects on the lung were predominantly due to its mucolytic property. Nevertheless, this belief is outdated, and more prominence has been given to NAC's anti-inflammatory effects [82]. The results of several studies have indicated that NAC reduces COPD exacerbations [83, 84], although further analysis of these data showed that this reduction was greatest in current smokers and patients not treated with inhaled corticosteroids [85]. The beneficial effect of NAC observed in several studies might correspond to the sum of these characteristics. Several studies also have addressed NAC's ability to relieve IPF. Despite encouraging results in animal models of fibrosis [86], NAC supplementation has not been highly effective in the clinic [87].

The membrane-bound complex NADPH oxidase (NOX) is a major source of ROS. In COPD and IPF, the principal cellular sources of ROS are NOXes and the mitochondria [88]. There are several isoforms of the catalytic component of NOX, including NOX1-5 and the dual oxidases DUOX1 and 2 [89]. Several NOX inhibitors have been developed to counteract oxidative stress [88]. Various studies indicate that NOX inhibitors may be beneficial in lung disease [90]. Apocynin is a nonselective NOX inhibitor; in cigarette-smoke-exposed mice, it reduced the levels of inflammatory cytokines and chemokines in bronchoalveolar fluid [91]. When administered by nebulization to COPD patients, apocynin reduced H_2_O_2_ and nitrite reduction in the exhaled breath condensate of COPD patients but no clinical parameters were reported [92]. Furthermore, recent studies have suggested that NOX4 is an important factor in the development of IPF, based on the enzyme's ability to induce alveolar epithelial cell death, (myo)fibroblast differentiation, and collagen deposition [93]. Setanaxib is a dual NOX1/4 inhibitor currently clinical development in an indication of IPF; it has demonstrated excellent tolerability and a reduction in various markers of chronic inflammation [94].

SODs are the only enzymes that can convert superoxide radicals to H_2_O_2_. There are three types of SOD: cytosolic copper-zinc SOD (cytosolic Cu/ZnSOD), mitochondrial manganese SOD (MnSOD), and extracellular SODs (ECSOD). In human studies, SOD activity in the bronchial epithelium, in the cells in bronchoalveolar fluid, and in bronchial brushings is lower in patients with asthma than in control subjects [95]. The role of SOD in the progression of IPF is less well understood. In fact, SOD1 is reportedly elevated in patients with IPF [96], and SOD1 knockout mice developed less oxidative stress and were protected from asbestos-induced pulmonary fibrosis, relative to wild-type littermates [97]. Although many previous antioxidant therapies have disappointed, newly characterized SOD mimetics appear to protect against oxidant-related lung disorders in animal models.

CAT is an antioxidant enzyme found almost in all living tissues that utilize oxygen. The enzyme uses either iron or manganese as a cofactor and catalyzes the degradation or reduction of hydrogen peroxide (H_2_O_2_) to water and molecular oxygen, consequently completing the dismutation reaction that occurs enzymatically by SOD [98]. Different lines of evidence have indicated that under inflammatory conditions, the levels of gene expression and the enzyme activities of CAT can be improved under treatment with metformin [99] [100]. Metformin, a biguanide derivate, is commonly used to treat patients with type 2 diabetes mellitus [101] and possesses its activities dependent of AMP-activated protein kinase (AMPK) [102]. It has been reported that AMPK activation acts via multiple mechanisms to reduce oxidative stress and is associated with increased levels of the antioxidant enzymes, including catalase [103]. Although the precise molecular mechanisms of AMPK have not been fully elucidated, there is cumulative evidence suggesting that AMPK activation protects against the development of emphysema and COPD by regulating Nrf2 activation [104].

GPx activity is significantly reduced in subjects with asthma or COPD that indicates its prominent role in lung antioxidant defense [105] [106]. In addition, there is a direct relationship between systemic GPx activity and FEV1 [107], and oxidative stress correlates with both lung function and body mass index in COPD [108]. Strategies to enhance the GPx-like activity have been used in the treatment of distinct pathological conditions, including COPD [109]. Ebselen is an organoselenium compound with hydroperoxide- and peroxynitrite-reducing activity that acts as an GPx mimetic being effective in reducing airway inflammation induced by ozone in rats [110] and inflammatory cytokines in the lungs of cigarette-smoke-exposed mice [91]. Ebselen has been used in clinical trials of acute ischemic stroke [111]; however, no studies have yet been reported on its protective role in asthma or COPD yet.

Myeloperoxidase is produced in neutrophils and macrophages. It has a damaging effect not only on bacteria but also on tissue. Thus, the selective, irreversible myeloperoxidase inhibitor 2-thioxanthine inactivated NF-*κ*B and reduced oxidative stress and the development of emphysema in guinea pigs exposed to cigarette smoke [112].

Antioxidant enzyme defense systems (including SOD, CAT, GPx, reduced glutathione, and heme oxygenase-1) are directly regulated by Nrf2. Thus, owing to its antioxidant effect, Nrf2 is a potential therapeutic target in lung disease [113]. Sulforaphane (a compound extracted from broccoli) was found to be a Nrf2 activator; experiments on human macrophages or mouse models suggest a preventive effect on COPD exacerbation [114]. Clinical studies have evidenced elevated Nrf2 expression in the lungs of patients with IPF [115]. However, further research on Nrf2 as a target in IPF treatment is needed.

### 4.2. Nonenzymatic Antioxidant Drugs

Dietary antioxidants (including vitamin C (ascorbic acid), vitamin E (*α*-tocopherol), resveratrol, and flavonoids) have been suggested as antioxidant treatments [116, 117]. The antioxidant and anti-inflammatory effects of these compounds have been demonstrated in the *in vitro* and *in vivo* model of inflammation induced by bleomycin, lipopolysaccharide, and cigarette smoke, among others [116]. Nevertheless, dietary antioxidant intake has not been shown to improve lung function or relieve clinical features in COPD. Furthermore, other researchers have shown that an antioxidant diet protects against emphysema but increases mortality in cigarette-smoke-exposed mice [117]—suggesting that the indiscriminate use of antioxidant dietary supplementation is even riskier. Unfortunately, large randomized clinical trials have yielded disappointing results, and recent meta-analyses concluded that indiscriminate, high-dose vitamin E supplementation results in increased mortality [118]. Indeed, we showed previously that supplementation with NAC and vitamin E was associated with elevated plasma levels of corticosterone in the rat [119].

### 4.3. Other Drugs That Affect Oxidative Stress

The treatments of COPD include oxygen supplementation, as well as oral, inhaled, or transdermal bronchodilators and/or inhaled corticosteroids [120]. These treatments may work together by affecting the redox imbalance. The results of clinical trials indicate that symptom relief alone might not be directly linked to a better prognosis. This is probably because treatment with a bronchodilator alone may fail to fully prevent ischemia; hence, ROS will still be generated and cause inflammation, due to the ischemic cascade or ischemia-reperfusion injury [121]. The disparities in the effects on symptoms and the prognosis suggested that oxygen supplementation has more direct disease-modifying action in COPD than bronchodilators do.

Pirfenidone is one of only two drugs approved by the US Food and Drug Administration in an indication of IPF. This compound is thought to have antioxidative, anti-inflammatory, and antifibrotic effects, although the exact mechanisms in IPF have not been clearly characterized. *In vivo* studies of bleomycin-induced murine pulmonary fibrosis indicate that pirfenidone reduces markers of oxidative stress, decreases the secretion of proinflammatory cytokines, and inhibits fibroblast proliferation, myofibroblast differentiation, and TGF-*β*-induced collagen production [122].

ROS may have a role (either directly or via the formation of lipid peroxidation products such 4-hydroxy-2-nonenal and F(2)-isoprostanes) in enhancing the inflammation through the activation of stress kinases (JNK, MAPK, p38, and PI3K) and thus increased activity of transcriptional factors such as NF-*κ*B, AP-1, and Nrf2. These enhanced intracellular signals are associated with the pathogenesis of COPD, IPF, and asthma. Thus, agents that modify these targets are the drug candidates for various lung diseases [123].

The association between corticosteroid resistance and PI3K inhibition was discussed above. Hence, treatment with a combination of a PI3K inhibitor and a corticosteroid should be a practical means of resolving inflammation in COPD. p38 MAPK inhibitors are capable of suppressing the release of proinflammatory mediators from alveolar macrophages and other immune/inflammatory cells taken from patients with COPD [124]. The dual p38*α*/*β* oral inhibitor losmapimod has also been investigated: it decreased the number of moderate-to-severe COPD exacerbations in patients with blood eosinophil counts ≤ 2% [125]. Furthermore, it was recently shown that the orally administered p38 MAPK inhibitor acumapimod decreased the number of hospital readmissions for COPD exacerbation [126]. Moreover, the p38*α*MAPK inhibitor can reinstate corticosteroid sensitivity in alveolar macrophages obtained from patients with asthma [58, 127]. Nevertheless, one should bear in mind that the p38 MAPK inhibitor has potential negative effects. In particular, abrogation of the physiologic functions exerted by p38 MAPK (notably with regard to innate immunity and antibacterial surveillance) could increase the patient's risk of infections, skin rash, and gastrointestinal, hepatic, cardiac, and central nervous system toxicity. Taken as a whole, these data suggest that there are still several barriers to the use of p38 MAPK inhibitors in IPF or COPD [128].

In the setting of chronic inflammatory lung disease, oxidative stress activates kinases and redox-sensitive transcription factors and modulates epigenetic chromatin modifications—resulting in changes in gene transcription. Recent studies have focused on identifying genes that undergo epigenetic modifications. In patients with asthma, microarray profiling of genes expressed in peripheral blood mononuclear cells can predict glucocorticoid sensitivity [129]. Novel means of circumventing steroid-refractory disease are currently being developed. Activation of HDAC2 and the reversal of oxidative posttranslational modifications of HDAC2 constitute other possible epigenetic-based therapeutic principles for severe asthma and COPD [130]. In the future, epigenetic profiling might be used to choose the best treatment option for lung disease [131]. However, the treatment of nonneoplastic lung diseases with epigenetic modifying drugs is in its infancy, with preclinical studies *in vitro* and *in vivo* models [132–134].

Another point to bear in mind is that although many studies have found that the accumulation of oxidative damage in cellular macromolecules is immensely toxic, the ROS produced by normal cell metabolism are vital for cellular homeostasis—especially for immune competence and the activation of several signal transduction pathways. Lastly, several different approaches to antioxidant treatment of lung disease have been explored *in vitro* and *in vivo* models but few have been clinically effective—perhaps because the oxidative stress (but not disease onset or progression) was affected in the preclinical studies. Nevertheless, today's knowledge of mechanisms of ROS regulation might lead to the pharmacological manipulation of antioxidants and the development of novel, truly effective drugs. The antioxidant treatment approach might provide a ray of hope in the otherwise difficult setting of COPD, asthma, and IPF. However, much work remains to be done.

## 5. Concluding Remarks

A growing body of evidence shows that oxidative imbalance has several pivotal roles in the pathophysiology of inflammatory lung diseases. Elevated ROS levels directly or indirectly affect a variety of receptors, other signaling molecules, proteins, and ion levels. The depletion of antioxidants and the accumulation of ROS reduce the cell's ability to mount an effective antioxidant response and thus contribute to the development of inflammatory lung and airway diseases. Therefore, a better understanding of the mechanisms through which the ROS affect intracellular homeostasis, cell signaling, and thus the onset and/or aggravation of inflammatory lung diseases may aid in the identification of new molecular pathways and the development of innovative, effective therapeutic strategies.

## Data Availability

This manuscript is a review. There is no original data.
